# Predicting biomarkers from classifier for liver metastasis of colorectal adenocarcinomas using machine learning models

**DOI:** 10.1002/cam4.3289

**Published:** 2020-07-24

**Authors:** Han Shuwen, Yang Xi, Zhou Qing, Zhuang Jing, Wu Wei

**Affiliations:** ^1^ Department of Oncology Huzhou Central Hospital Affiliated Central Hospital Huzhou University Huzhou China; ^2^ Department of Oncology Huzhou Central Hospital Affiliated Central Hospital Huzhou University Huzhou China; ^3^ Department of Nursing Huzhou Central Hospital Affiliated Central Hospital Huzhou University Huzhou China; ^4^ Graduate School of Nursing Huzhou university Huzhou China; ^5^ Department of Gastroenterology Huzhou Central Hospital Affiliated Central Hospital Huzhou University Huzhou China

**Keywords:** CatBoost algorithm, colorectal adenocarcinomas, feature genes, liver metastasis, machine learning approaches

## Abstract

**Background:**

Early diagnosis of liver metastasis is of great importance for enhancing the survival of colorectal adenocarcinoma (CAD) patients, and the combined use of a single biomarker in a classier model has shown great improvement in predicting the metastasis of several types of cancers. However, it is little reported for CAD. This study therefore aimed to screen an optimal classier model of CAD with liver metastasis and explore the metastatic mechanisms of genes when applying this classier model.

**Methods:**

The differentially expressed genes between primary CAD samples and CAD with metastasis samples were screened from the Moffitt Cancer Center (MCC) dataset GSE131418. The classification performances of six selected algorithms, namely, LR, RF, SVM, GBDT, NN, and CatBoost, for classification of CAD with liver metastasis samples were compared using the MCC dataset GSE131418 by detecting their classification test accuracy. In addition, the consortium datasets of GSE131418 and GSE81558 were used as internal and external validation sets to screen the optimal method. Subsequently, functional analyses and a drug‐targeted network construction of the feature genes when applying the optimal method were conducted.

**Results:**

The optimal CatBoost model with the highest accuracy of 99%, and an area under the curve of 1, was screened, which consisted of 33 feature genes. A functional analysis showed that the feature genes were closely associated with a “steroid metabolic process” and “lipoprotein particle receptor binding” (eg APOB and APOC3). In addition, the feature genes were significantly enriched in the “complement and coagulation cascade” pathways (eg FGA, F2, and F9). In a drug‐target interaction network, F2 and F9 were predicted as targets of menadione.

**Conclusion:**

The CatBoost model constructed using 33 feature genes showed the optimal classification performance for identifying CAD with liver metastasis.

## Graphical abstract

APOB, APOC3, FGA, F2, F9, and NKX2‐3 were potential biomarkers for classification of CAD with liver metastasis. Menadione might be a promising anti‐metastatic drug of CAD cells through functioning its role at sites of F2 and F9. CatBoost model constructed by 33 feature genes showed the optimal classification performance for identifying CAD liver metastasis.

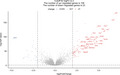

## 1 INTRODUCTION

Colorectal cancer (CRC) was the second‐leading cause of cancer mortality worldwide in 2018, just behind lung cancer, and was the fifth most common cause of cancer deaths in China, the trend of which is rising.^1^ Changes in bowel habits and the occurrence of stomachaches and bloody stools are the main clinical manifestations of CRC.^2^ Colorectal adenocarcinoma (CAD), which originates from the epithelial cells of the colorectal mucosa, accounts for 90% of the occurrences of CRC.^3^ It has been found that for more than 20% of patients CAD may metastasize.^4^ Liver metastasis is the poorest prognostic factor of CAD, and the resection rate for colorectal liver metastasis remains at less than 25%.^5^ Thus, the early diagnosis of liver metastasis is extremely important for enhancing the survival of CAD patients.

Methods for an early detection of CAD with liver metastasis are lacking. A new method for analyzing the transcriptomic differences between primary CAD and a distant metastasis was developed, and FBN2 and MMP3 were identified as CAD metastasis related genes, which may help predict a high‐level risk of CAD metastasis.^4^ Sayagués et al revealed the existence of several dysregulated genes including APOA1, HRG, UGT2B4, and RBP4 in CAD with liver metastasis samples in comparison to the primary tumor.^6^ Qian et al identified higher expressions of THBS2, INHBB, and BGN in CRC patients with liver metastasis.^7^ However, a single biomarker has generally shown no advantages in the prediction and classification of cancer metastasis samples over a combination of biomarkers.

Machine learning a computer‐based algorithm, has shown high degrees of accuracy and prediction that exceeds the abilities of standard statistical methods to make predictions about outcomes in patients.^8^ Machine learning approaches applying different datasets have recently been proposed to improve the classification of primary cancers and metastasis samples, as well as to predict cancer metastasis.^9^, ^10^ Tapak L *et al* have found that the random forest (RF) has the highest specificity, the Naive Bayes (NB) has highest sensitivity while the traditional machine learning approaches [logistic regression (LR) and linear discriminant analysis] had the highest total accuracy for metastasis prediction in breast cancer.^11^ In addition, the support vector machine (SVM) outperformed other machine learning methods for breast cancer survival prediction.^11^ Montazeri et al have demonstrated Trees Random Forest model (TRF) has highest level of accuracy for survival prediction in breast cancer than SVM, NB, 1‐Nearest Neighbor (1NN) and Multilayer Perceptron (MLP).^12^ The results of different studies find different methods as the most reliable one for disease prediction and it is inconsistency about the results comparisons of various machine learning algorithms in the classification accuracy of data mining for disease prediction.

Although machine learning techniques comparisons are widely studied in cancer metastasis such as breast cancer and nonsmall cell lung cancer,^11^, ^13^ there was little on CAD metastasis. In our study, six machine learning approaches (LR,^14^ RF,^15^ SVM, gradient boosting decision tree (GBDT),^16^ neural network (NN),^17^ and categorical boosting (CatBoost) ^18^) were applied to construct prediction models for a CAD liver metastasis by CAD metastasis related‐differentially expressed genes (DEGs). Then, the classification accuracy of six models were analyzed in training set, and the classification performances of classier models were validated to screen the optimal method. Subsequently, functional analyses of feature genes were conducted using this optimal method. Finally, the protein‐protein interaction (PPI) network and drug‐target interaction network of feature genes were constructed (Figure S1). Thus, this study is aimed at screening the feature genes classified as potential biomarkers when applying the optimal method, and comprehensively evaluating the metastatic mechanisms and treatment targets of CAD with liver metastasis.

## 2 MATERIALS AND METHODS

### 2.1 Data source

Two datasets, GSE131418 and GSE81558, downloaded from Gene Expression Omnibus (GEO) were used in this study. The Moffitt Cancer Center (MCC) dataset GSE131418 was used as the training set, whereas the consortium datasets GSE131418 and GSE81558 were used as internal and external validation sets for the liver metastasis models respectively. GSE131418 includes 333 CAD and 184 liver metastasis samples from the MCC cohort dataset, and 545 primary CAD and 73 liver metastasis samples from a consortium cohort dataset. The transcriptomic data of GSE131418 were generated from the GPL15048 Rosetta/Merck Human RSTA Custom Affymetrix 2.0 microarray platform [HuRSTA_2a520709.CDF]. In addition, a total of 23 primary CAD and 19 liver metastasis samples were analyzed from GSE81558. The sequencing platform of GSE81558 was the GPL15207 [PrimeView] Affymetrix Human Gene Expression Array.

### 2.2 Data preprocessing

Before data preprocessing, GSE131418_RAW.tar was downloaded from GEO using the GEOquery package.^19^ A series of processes including a background correction, normalization, and calculation of the genes expressions were conducted for the microarray data using the affy package in R.^20^ Later, the annotation files were downloaded and the probe ID was converted into the gene symbol. The probes without corresponding gene symbols were deleted, and the mean of the probes mapped to the same gene symbol were calculated as the expression value of this gene. For GSE81558, the expression data of the downloaded Series Matrix File(s) were standardized using the robust multiarray average (RMA) algorithm. Subsequently, a principal component analysis (PCA) was conducted to observe the sample grouping by the FactoMineR package in R.

### 2.3 Screening of DEGs and hierarchical clustering

A modified t‐test applying an empirical Bayesian method was applied to conduct mRNA transcriptomic differences between the primary CAD and CAD with liver metastasis groups. The DEGs were then identified under *P*‐value < 0.05 and |log_2_ fold change (FC)|> 2. In addition, the ggscatter function of the ggpubr package in R was used to draw a volcano plot of the DEGs, and the gene symbols of the top‐30 DEGs ranked by |log_2_ FC| were presented. The pheatmap package in R was applied to conduct the hierarchical clustering.

### 2.4 Construction of liver metastasis prediction models

The count data of the DEGs were transformed into log2(x + 1) formatted data, and a binary label value of “1” was used for classifying the liver metastasis samples, and a value of “0” was used for classifying the nonmetastasis samples. For each group, 80% of the samples were divided into a training set using the train_test_split machine learning method in Python (version 0.21.2),^21^ whereas 20% of the samples were divided into the test set.

Before the model construction, the recursive feature elimination (RFE) algorithm based on the sklearn.feature_selection method was applied to the feature selection. Machine learning models, ie LR, based on the sklearn.linear_model; RF and GBDT, based on sklearn.ensemble; an SVM, based on sklearn.svm; and NN, based on sklearn.neural_network, were constructed (Data S1). Another CatBoost machine learning model was constructed using the Catboost package (version 0.16.5).^22^

### 2.5 Validation of prediction models and screening of optimal model

The consortium datasets GSE131418 and GSE81558 were used as the internal and external validation sets for the predicted models above respectively. First, the feature DEGs were input into the six well‐trained or constructed liver metastasis models described above. The expression values of the feature DEGs in the samples were utilized as an eigenvalue to classify and identify CAD samples with or without liver metastasis. The risk of liver metastasis in the samples of the validation sets was predicted by assessing the accuracy and AUC values, which were used to evaluate the prediction and classification capability of the six models.

Following the construction and data validation of the six models, the model with the highest AUC value in both the training and validation sets was screened as the optimal model. The feature genes in the optimal model were chosen for the following analysis.

### 2.6 Functional enrichment analysis of feature genes

Kyoto Encyclopedia of Genes and Genomes (KEGG) is a database offering a biological interpretation of the genome function through KEGG pathway mapping, and links genomic information to more ordered information of biological functions.^23^ Gene Ontology (GO) is a annotation database supplying gene functions from three ontologies, namely, the biological process (BP), cellular component (CC), and molecular function (MF) ontologies.^24^ In this study, the clusterProfiler tool (version 3.12.0) was used to conduct the GO terms and KEGG pathway enrichment analysis.^25^ The significant enrichment results were chosen with a cut‐off of *P* value < 0.05 and count ≥ 2.

### 2.7 Construction of PPI network

The Search Tool for the Retrieval of Interacting Genes (STRING) database provides available sources on protein‐protein associations for 5,090 organisms.^26^ In our study, STRING (version 11.0, http://www.string‐db.org/) was used to predict interactions of the feature genes with a PPI score of 0.4 (medium confidence), disable structural previews inside network bubbles, and hide disconnected nodes in the network. The pairs obtained were then visualized in a PPI network using Cytoscape software.^27^ In addition, subnetwork mining of the PPI network was conducted by applying MCODE under a degree cut‐off of 2, node score cut‐off of 0.2, K‐core of 2, and depth from seed of 100 as the parameters.

### 2.8 Drug target prediction of feature genes

The drug‐gene interaction database is an open resource used to excavate information of a drug‐gene interaction and druggable genome.^28^ In our study, DGIdb 3.0 (www.dgidb.org) was used to predict the small drug molecules that interact with the feature genes. The drug‐target interactions reported in previous papers and by the FDA were then obtained. Finally, the drug‐target interaction network was constructed using Cytoscape.

## 3 RESULTS

### 3.1 Basic statistical information of GSE131418 and GSE81558

Information regarding the total number of samples, the probes, the annotated probes, and the gene symbols of GSE131418 and GSE81558 are shown in Table 1. There were 60 607 and 49 395 probes in the raw expression matrix of GSE131418 and GSE81558 respectively. After annotation, a total of 47 408 probes involved in 24 495 genes were obtained from the GSE131418 dataset, and a set of 46 879 probes related to 18 835 genes were obtained from the GSE81558 dataset.

**TABLE 1:** The basic information of each dataset

Information	GSE131418	GSE81558
Total samples	1135	51
MCC/Consortium	517/618	/
Total probe	60 607	49 395
Annotated probe	47 408	46 879
Corresponding gene symbol	24 495	18 835
Primary/Liver metastases (total)	333 + 545/141 + 56/(878/197)	23/19

Abbreviation: MCC: Moffitt Cancer Center.

After data preprocessing, boxplots of the normalized expression values and PCA plots of the samples in the MCC cohort (Figure S2), CON cohort (Figure S3), and GSE81558 (Figure S4) datasets are drawn. The black lines in the middle of each of these boxplots are nearly straight, indicating that the data are normalized well. In addition, a PCA analysis of the samples showed that different groups exhibit partial differences, but without a significant batch effect. These indicate that preprocessed data are suitable for the following analysis.

### 3.2 DEGs screened between two groups

**FIGURE 1:**
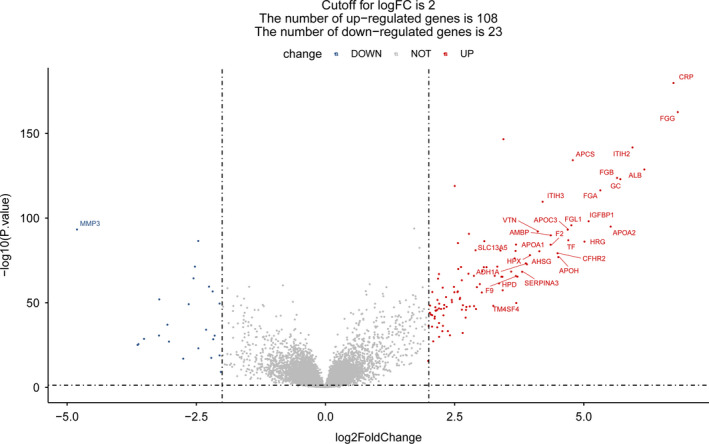
Volcano plot of differentially expressed genes (DEGs) in MCC cohort dataset. The volcano plot was drawn using the ggpubr package. The gene symbols of the top‐30 DEGs ranked by |log_2_ FC| are presented. A total of 268 DEGs involving 108 upregulated DEGs and 23 downregulated DEGs, and 24,364 non‐DEGs between primary CAD and liver metastasis samples. The X‐axis represents the change in fold of the genes, and the Y‐axis represents the p value. The red square represents upregulated DEGs, the blue circle represents downregulated DEGs, and the black triangle represents nondifferential genes

**FIGURE 2:**
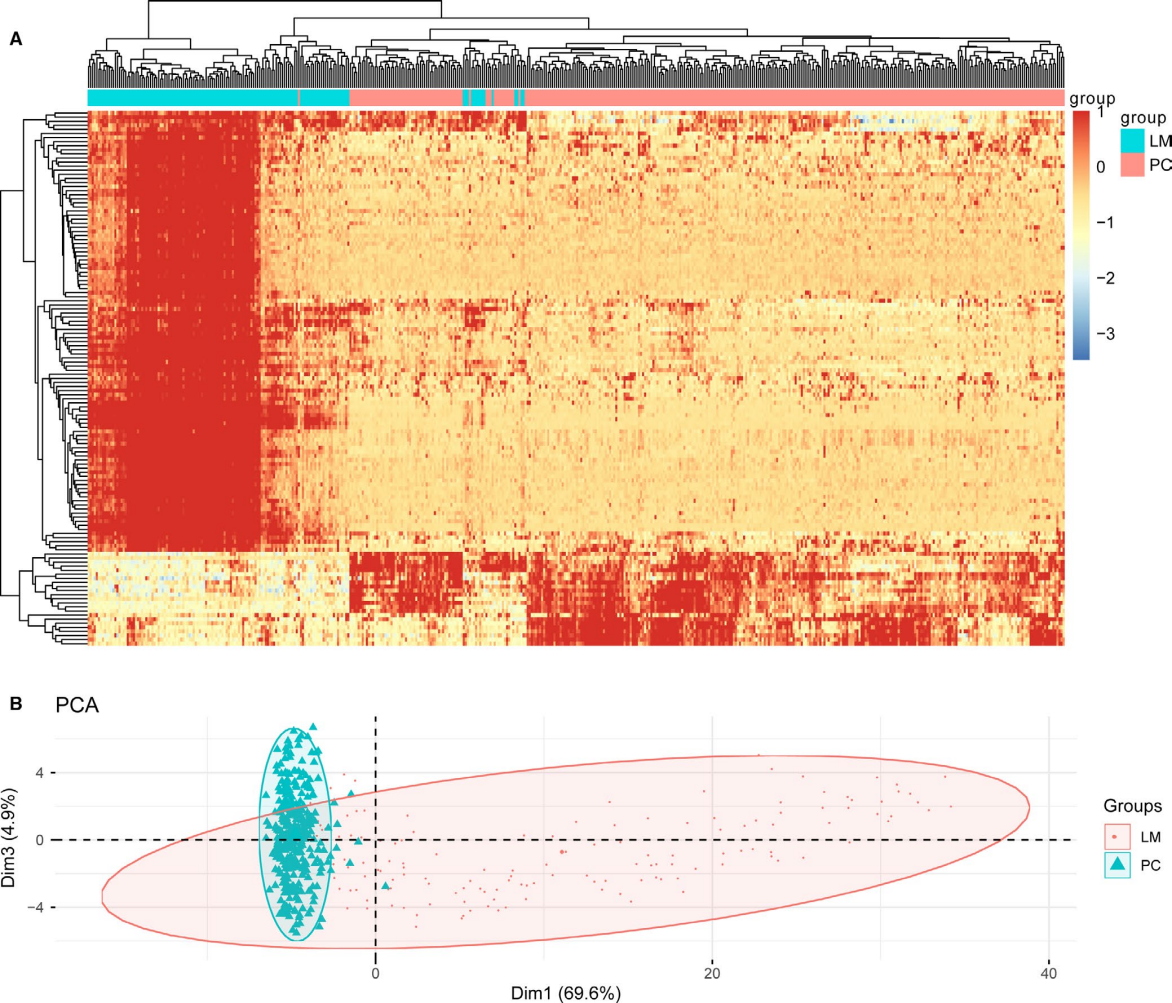
Clustergrams of DEGs in primary CAD and liver metastasis samples. (A) The pheatmap of DEGs. The pheatmap package in R was applied to conduct hierarchical clustering. (B) PCA plot of DEGs drawn using the FactoMineR package in R. In the PCA plot, Dim 1 is presented on the x‐axis and Dim 3 is presented on the y‐axis. The cluster heatmap and PCA plot of the DEGs showed a good discrimination among the primary CAD and liver metastasis samples. Red indicates upregulated DEGs, whereas blue indicates upregulated DEGs. LM, liver metastasis; PC, primary cancer

Under the threshold of *P* value < 0.05 and |log_2_FC|> 2, a total of 268 DEGs involving 108 upregulated DEGs and 23 downregulated DEGs were identified, whereas the expressions of 24 364 genes were not significantly changed between the primary CAD sample and CAD with liver metastasis samples in the MCC cohort dataset (Figure 1). The cluster heatmap (Figure 2A) and PCA plot (Figure 2B) of the DEGs demonstrated a good discrimination among the primary CAD and liver metastasis samples.

### 3.3 Performance evaluation outcomes and validation results

**FIGURE 3:**
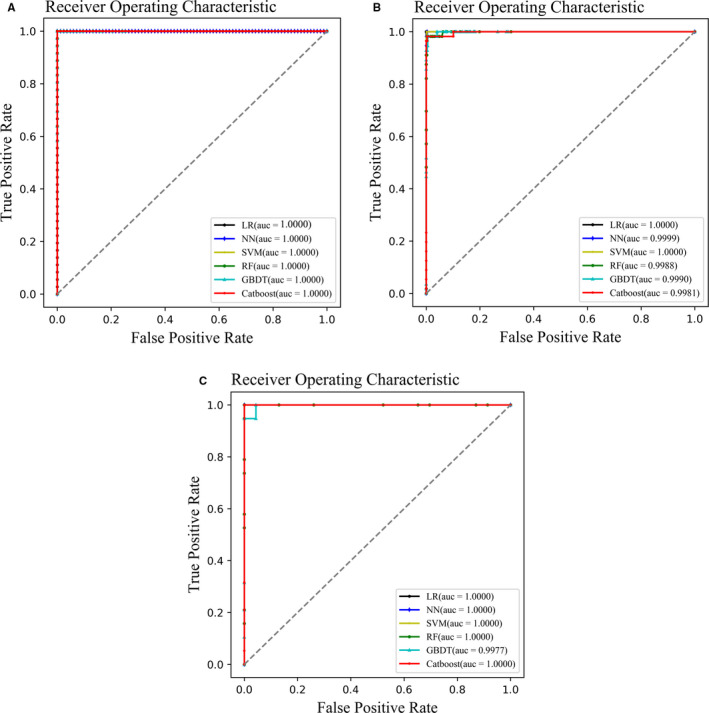
ROC curves of six models. (A) ROC curves of six models constructed using the training set. (B) ROC curves of six models constructed using internal validation set. (C) ROC curves of six models constructed using external validation sets. The dashed grey line represents a line of equality or random chance. ROC, receiver operating characteristic

The numbers of feature DEGs, the accuracy, and the AUC of the ROC values of six models based on data from the training set, and internal and external validation sets, are presented in Table 2. The accuracy of each model in the training set ranged from 0.983193 to 1, and the AUC of each model reached up to 1 (Figure 3A). In the internal validation sets, the accuracy and AUC of each model were similar (Figure 3B). In the external validation sets, the accuracy and AUC of the NN and Catboost models all reached up to 1 (Figure 3C). Overall, the accuracy and AUC of the NN and Catboost models for the different datasets were relatively higher than those of the other models. However, we failed to use an NN to screen the feature genes because all DEGs were input into this model. Thus, the Catboost model was considered optimal.

**TABLE 2:** The values of the classification performance of six machine learning models for predicting liver metastasis of the colorectal adenocarcinomas

Models	Features	Accuracy (MCC)	AUC (MCC)	Accuracy (Con)	AUC (Con)	Accuracy (GSE81558)	AUC (GSE81558)
LR	25	1	1	1	1	0.97619	1
NN	131	1	1	0.996672	0.999934	1	1
SVM	131	0.991597	1	0.996672	0.999967	0.97619	1
RF	21	0.991597	1	0.995008	0.998755	0.97619	1
GBDT	38	0.983193	1	0.993344	0.999017	0.97619	0.997712
Catboost	33	0.991597	1	0.993344	0.998132	1	1

Note: The MCC Cohort of GSE131418 was used as training set, while Consortium Cohort of GSE131418 and GSE81558 were used as internal and external validation sets for liver metastasis models, respectively.

Abbreviation: LR: logistic regression; NN: neural network; SVM: support vector machine; RF: random forest; GBDT: gradient boosting decision tree; Catboost: categorical boosting; MCC: Moffitt Cancer Center; con: Consortium.

### 3.4 Optimal Catboost model

**FIGURE 4:**
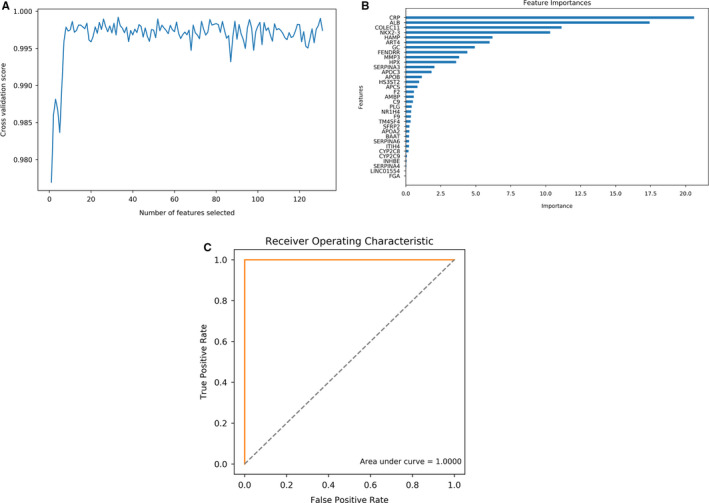
Relevant outcomes of optimal Catboost model. (A) Line chart of RFE algorithm used to screen the feature genes under different cross‐validation scores, and a total of 33 feature genes were obtained with the highest cross‐validation score. The X‐axis represents the numbers of selected feature genes, and the Y‐axis represents cross‐validation scores. (B) Importance assessment of 33 feature genes. (C) ROC curve of the Catboost model. The AUC is 1. ROC, receiver operating characteristic

In the Catboost model, the RFE algorithm was used to screen the feature genes under different cross‐validation scores, and a total of 33 feature genes were obtained with the highest cross‐validation score (Figure 4A). The importance of these feature genes was evaluated, and CRP, ALB, COLEC11, NKX2‐3, HAMP, ART4, and GC showed a higher importance than the other genes (Figure 4B). The AUC of this model reached up to 1, which may be associated with the significant difference between the primary CAD samples and CAD with liver metastasis samples (Figure 4C).

### 3.5 Functions of 33 feature genes

**FIGURE 5:**
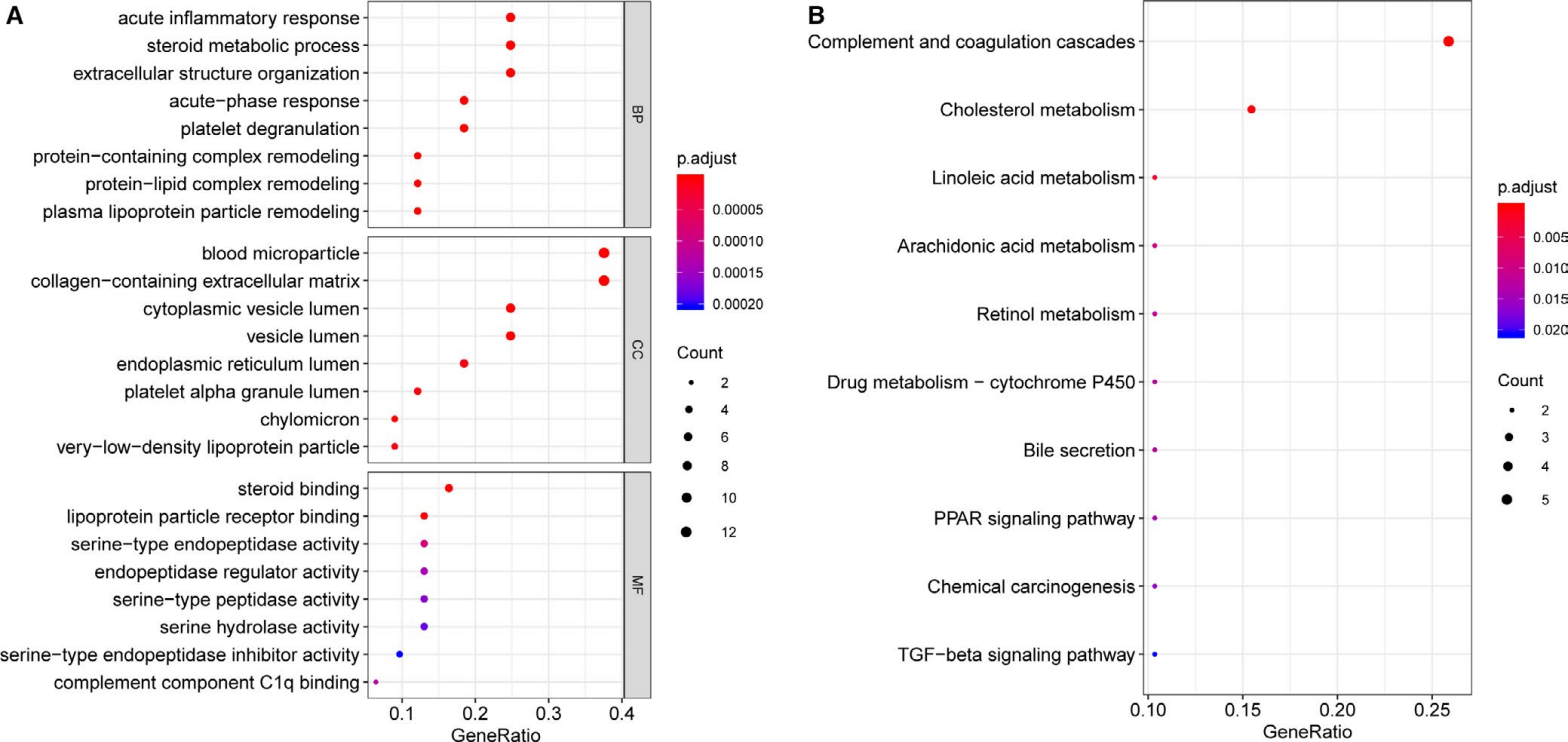
Functional enrichment analyses of feature genes in optimal Catboost model. (A) Bubble diagram of GO enrichment result. The clusterProfiler tool (version 3.12.0) was applied to analyze GO terms. Top‐8 enriched terms of GO BP, CC, and MF are presented. (B) KEGG enrichment analyses results. The top‐10 enriched KEGG pathways are presented. Significant enrichment results were chosen based on a cut‐off of P value < 0.05 and count ≥ 2. The size of the dot represents the proportion of genes, which is positively associated with the proportion of corresponding enrichment items. The change in color from dark blue to red represents a change in p value from low to high. GO, Gene ontology; KEGG, Kyoto Encyclopedia of Genes and Genomes; BP, biological process; CC, cellular component; MF, molecular function

The analysis results of the GO terms showed that the feature genes were most significantly associated with the BP of “acute inflammatory response” (GO:0 002 526), CC of “blood microparticle” (GO:0 072 562), and MF of “steroid binding” (GO:0 005 496, e.g., APOB and APOC3) (Figure 5A). Notably, most of the top‐8 enriched terms of GO BP, CC, and MF were associated with a lipid metabolic process, such as the “steroid metabolic process” (e.g., APOB and APOC3) and “lipoprotein particle receptor binding” (e.g., APOB and APOC3). In addition, the feature genes were significantly enriched in the “complement and coagulation cascades” (e.g., FGA, F2, and F9) pathway (Figure 5B).

### 3.6 PPI network and subnetwork of feature genes

**FIGURE 6:**
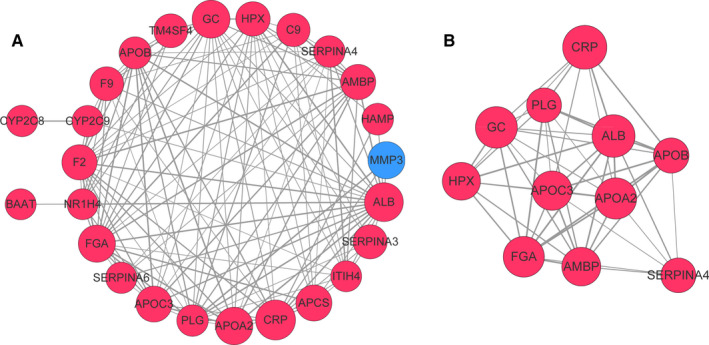
Interactions of feature genes in optimal Catboost model presented in a network. (A) Protein‐protein interaction (PPI) network. (B) Top sub‐network from PPI network. The upregulated and downregulated genes are indicated by red and blue respectively. PPI network for interactions of DEGs with confidence score of > 0.4

When setting the minimum interaction scores as 0.4, only 25 of the 33 feature genes had interactions with pairs of the other genes (Figure 6A). Thus, the PPI network consists of these 25 feature genes and 128 PPI pairs. The majority genes (24) in the PPI network were upregulated and only one gene (MMP3) was downregulated. The top‐10 nodes in the PPI network with a high degree were ALB, FGA, F2, GC, PLG, CRP, HPX, AMBP, APOC3, and APOB (Table 3). In addition, two subnetworks were obtained from the PPI network, and the one with higher score (9.6) is presented in Figure 6B. There were 11 nodes and 48 PPI pairs in this subnetwork, and all genes were upregulated.

**TABLE 3:** The nodes in PPI network ranked by degrees

Nodes	Degree	Betweenness	Closeness
ALB	20.0	98.05642	0.85714287
FGA	17.0	29.657936	0.75
F2	17.0	38.40642	0.7741935
GC	16.0	25.370707	0.72727275
PLG	15.0	18.715944	0.6857143
CRP	14.0	22.459524	0.6666667
HPX	14.0	15.992136	0.6666667
AMBP	13.0	5.752381	0.6486486
APOC3	13.0	9.283405	0.6666667
APOB	13.0	11.427056	0.6666667
ITIH4	12.0	6.084199	0.6315789
APOA2	12.0	2.9199135	0.6315789
SERPINA4	11.0	5.1714287	0.61538464
F9	11.0	17.425468	0.6486486
NR1H4	10.0	54.25	0.6315789
C9	9.0	2.8238096	0.58536583
APCS	9.0	2.2032468	0.58536583
SERPINA3	6.0	0.0	0.54545456
SERPINA6	6.0	0.0	0.54545456
TM4SF4	5.0	0.0	0.5217391
CYP2C9	5.0	46.0	0.55813956
HAMP	3.0	0.0	0.5
MMP3	3.0	0.0	0.5
BAAT	1.0	0.0	0.39344263
CYP2C8	1.0	0.0	0.36363637

### 3.7 Predicted drug targets of feature genes

**FIGURE 7:**
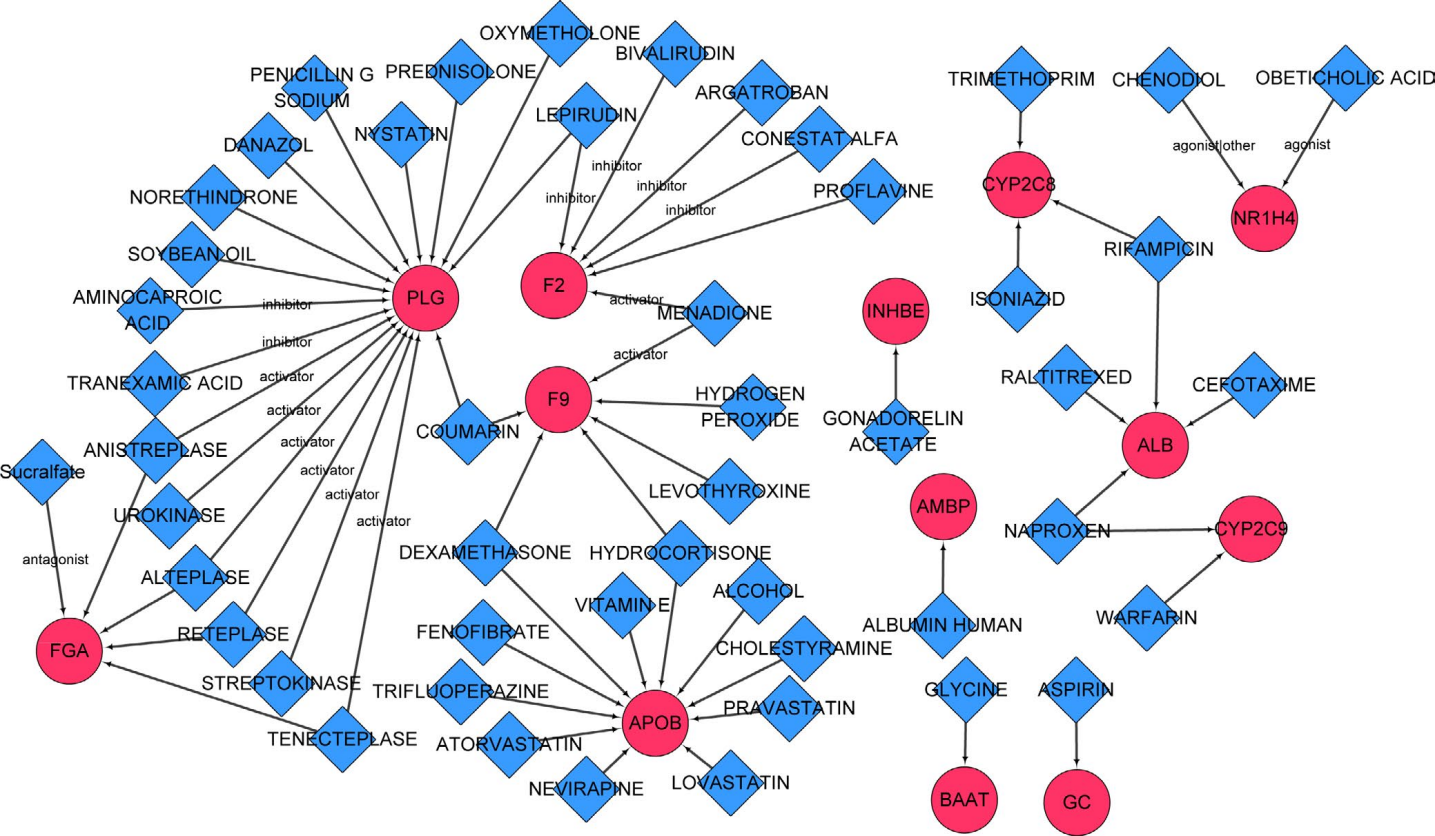
Drug‐target interaction network. DGIdb 3.0 was used to predict the small drug molecules that interact with the feature genes. The blue diamond represents the drug and the red circle represents a gene

Among the 33 feature genes, 31 genes were predicted to have drug binding sites. In total, a set of 254 drug‐gene pairs were identified, in which 125 were obtained from FDA‐approved drugs and 60 were obtained from published papers. Specifically, there were 13 feature genes predicted as targets of FDA‐approved drugs, or of drugs reported in previous studies. The drug‐gene pairs involving these 13 feature genes are presented in a drug‐target interaction network (Figure 7), in which F2 and F9 as coagulation factor family members were predicted as menadione targets.

## 4 DISCUSSION

In this study, we compared six selected algorithms (LR, RF, SVM, GBDT, NN, and CatBoost) to create classifiers for CAD classification with liver metastasis samples. The optimal model with the highest accuracy (99%) and AUC (1) based on the CatBoost algorithm was screened, and consists of 33 feature genes. A functional analysis showed that the feature genes were closely associated with a lipid metabolic process such as the “steroid metabolic process” (eg APOB and APOC3) and “lipoprotein particle receptor binding” (eg APOB and APOC3). In addition, the feature genes were significantly enriched in the “complement and coagulation cascade” pathway (eg FGA, F2, and F9), revealing the potential biomarkers and pathogenesis of CAD with liver metastasis.

According to the comparisons among six classification algorithms, the optimal model CatBoost was achieved for identifying CAD liver metastases with higher classification performance in training set and best reproducibility in validation set. Although NN classier showed a higher or equal classification performance in training, internal and external validation sets than CatBoost classier, the feature genes in this classier were consisted by all the DEGs (131), while there were 33 feature genes in CatBoost classier, which meant that the classification ability of 33 feature genes in CatBoost was almost equal to 131 feature genes in NN. The number of feature gene is also a critical parameter to evaluate the performance of classier, and a method with minimum number of feature genes for a classification problem with an objective function to maximize the classification accuracy is always needed.^29^ In similar way, SVM classier with 131 feature genes and lower accuracy in external validation set than CatBoost classier was excluded. Futuremore, the LR, RF, and Catboost classier with lower accuracy in external validation set than CatBoost classier were also excluded.

Except for the number of feature gene, the parameters of accuracy and AUC were also used to measure the performance of classier in this study. Accuracy may be interpreted as the proportion of instances the classifier always classify correctly for an given dataset or other data.^30^ The AUC of a classifier is a portion of the area of the unit square and has an good statistical property that the classifier will rank a randomly selected positive instance higher than a randomly selected negative instance.^31^ Consistently, most researchers have applied the combinations of accuracy and AUC to assess predictive ability of classifiers.^32^, ^33^

CatBoost is a new developed algorithm based on GBDT algorithm that can successfully handle categorical features with advantage of reducing overfitting on available datasets, and outperforms traditional GBDT algorithm to overcome the gradient bias with ordered boosting.^18^ CatBoost also outperforms other classifiers over different evaluation metrics in different analysis purpose.^34^ A study has indicated that CatBoost outperforms other machine learning classifiers LR, NB, RF, and SVM for anxiety and depression prediction, with an higher accuracy (82.6%) and precision and (84.1%).^35^ These findings were in line with our findings. However, it is important to note that CatBoost will not work best on all supervised classification problems.^36^

In addition, the underlying biological meaning of 33 feature genes in CatBoost classier was analyzed. In our study, we predicted that APOB and APOC3 were both upregulated in CAD liver metastasis samples, and associated with “steroid metabolic process” and “lipoprotein particle receptor binding”. Serum lipids are risk factors of numerous types of cancers, and has crucial roles in cancer metabolism.^37^, ^38^ Apolipoprotein B (APOB) as a lipid binding protein is a main component of chylomicrons and low‐density lipoproteins (LDL).^39^ ApoB‐100, as an isoform of APOB synthesized exclusively in the liver, is required for the production of triglyceride‐rich VLDL.^40^ Apolipoprotein C3 (APOC3) is a glycoprotein secreted by the liver and intestines, the expression of which is positively related to energy expenditure and energy demand by participating in the plasma triglyceride metabolism.^41^ Similarly, the increasing quartiles of ApoB‐100 and triglycerides are positively associated with the risk of CRC.^42^ In addition, the increased APOB/APOA1 ratio is related to the nodal metastasis of CRC.^43^ A rewiring of the lipid metabolic programs is necessary for cancer cells to acquire more nutrients and energy, and finally survive and develop metastases from the primary tumor.^44^ Thus, we speculated that APOB and APOC3 are potential biomarkers for classification of CAD with metastasis and without liver metastasis.

Coagulation factor II (F2, FII) or prothrombin has a pivotal role in maintaining the vascular integrity by regulating the thrombin using prothrombinase.^45^ Coagulation factor IX (F9, FIX) as a vitamin K‐dependent glycoprotein is a precursor of a serine protease.^46^ A fibrinogen alpha chain (FGA) encodes the alpha subunit of the fibrinogen. It has been indicated that the levels of fibrinogen and FIX are significantly higher in nonmetastatic CRC patients, and are considered as a risk factor for venous thromboembolism, whereas the expression of FII does not show a significant difference between nonmetastatic CRC and the controls.^47^ In our study, we found FGA, F2, and F9 to be significantly upregulated in CAD with liver metastasis. Although no related studies regarding FGA, F2, and F9 in CAD with liver metastasis have been reported, prothrombin (F2) expression is increased in CAD patients in comparison to normal patients.^48^ Notably, FVII, being in the same family as FII and FIX, was found to be upregulated in CRC with liver metastasis in comparison with nonmetastasis CRC.^49^ In addition, thrombin‐induced pro‐coagulant roles can enhance the metastatic potential of cancer cells.^50^ Meanwhile, an overexpression of fibrinogen is responsible for the liver metastasis of CRC, and a fibrinogen beta chain (FGB) is a diagnostic and therapeutic biomarker of CRC with liver metastasis.^51^ Thus, we inferred that FGA, F2, and F9 might be novel biomarkers for identification of CAD with liver metastasis.

NK2 Homeobox 3 (NKX2‐3) encodes a homeodomain‐containing transcription factor and as a member of the Nkx family is applied to determine the tissue differentiation.^52^ In a previous study, NKX2‐3 was screened as a new tumor suppressor of CRC.^53^ Yu et al later found that NKX2‐3 is downregulated in inflammatory bowel‐disease‐related CRC and might be involved in the development of CRC by regulating the Wnt signaling pathway.^54^ In addition, the reduced expression of Nkx2.8 is detected in invasive bladder cancer cells while enhancing the cell proliferation.^55^ Similarly, we found that NKX2‐3 is downregulated in CAD with liver metastasis. However, few studies on NKX2‐3 regarding the liver metastasis of various cancers have been reported. Thus, we suggest that NKX2‐3 might be a potential biomarker for the classification of CAD with or without liver metastasis.

Although several feature genes for predicting CAD with liver metastasis were screened in this study, and a functional analysis and drug prediction of these genes were conducted, the experimental verifications of these findings remain lacking. Thus, future research is required.

In conclusion, the CatBoost model showed the optimal classification performance in identifying CAD with liver metastasis. The feature genes in the CatBoost model, such as APOB, APOC3, FGA, F2, F9, and NKX2‐3 were demonstrated to be potential biomarkers for the classification and prediction of CAD with liver metastasis samples.

## 5 COMPETING INTERESTS

The authors declare that no conflicts of interest exist.

## AUTHORS’ CONTRIBUTIONS

All authors participated in the conception and design of the study; Conceived the manuscript: Han Shuwen and Yang Xi; Wrote the paper: Han Shuwen, Yang Xi, and Zhuang Jing; Processed the data: Wu Wei and Liu Jin; Drew figures: Zhuang Jing; All authors read and approved the paper.

## 6 ETHICS APPROVAL AND CONSENT TO PARTICIPATE

Not Applicable.

## 7 CONSENT FOR PUBLICATION

Not applicable.

## 8 AVAILABILITY OF DATA AND MATERIALS

The datasets generated during the current study are not publicly available but obtained from corresponding authors on reasonable request.

## Supporting information

Figure S1.

Figure S2.

Figure S3.

Figure S4.

Data S1.
