# Supplementary figures and images for: Predicting biomarkers from classifier for liver metastasis of colorectal adenocarcinomas using machine learning models
Source: Cancer Med. 2020 Jul 24;9(18):6667–78. doi: 10.1002/cam4.3289 (PMC7520257; doi:10.1002/cam4.3289)

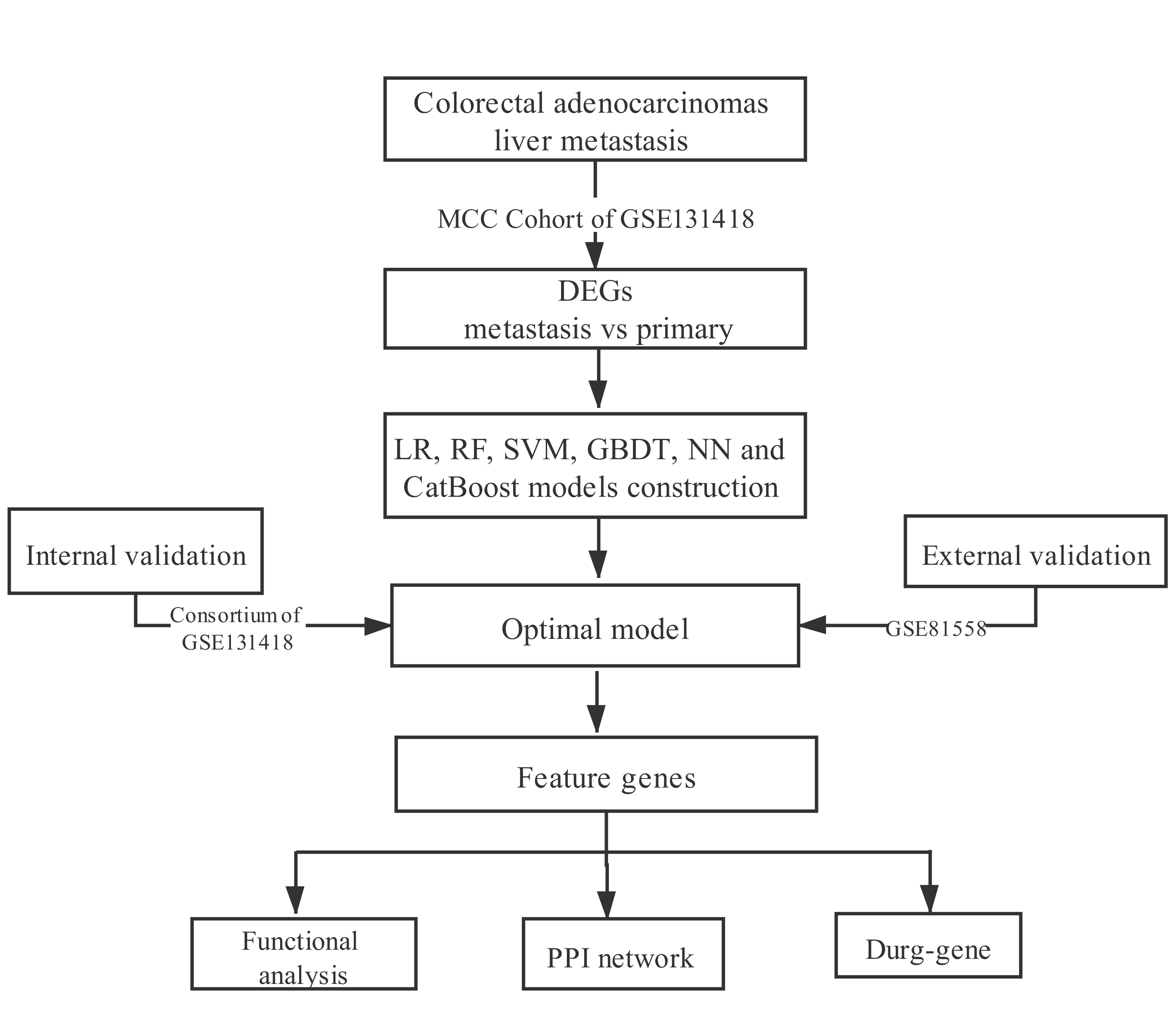

Supplement: Supplementary file 1 — Figure S1. [file CAM4-9-6667-s001.tif]

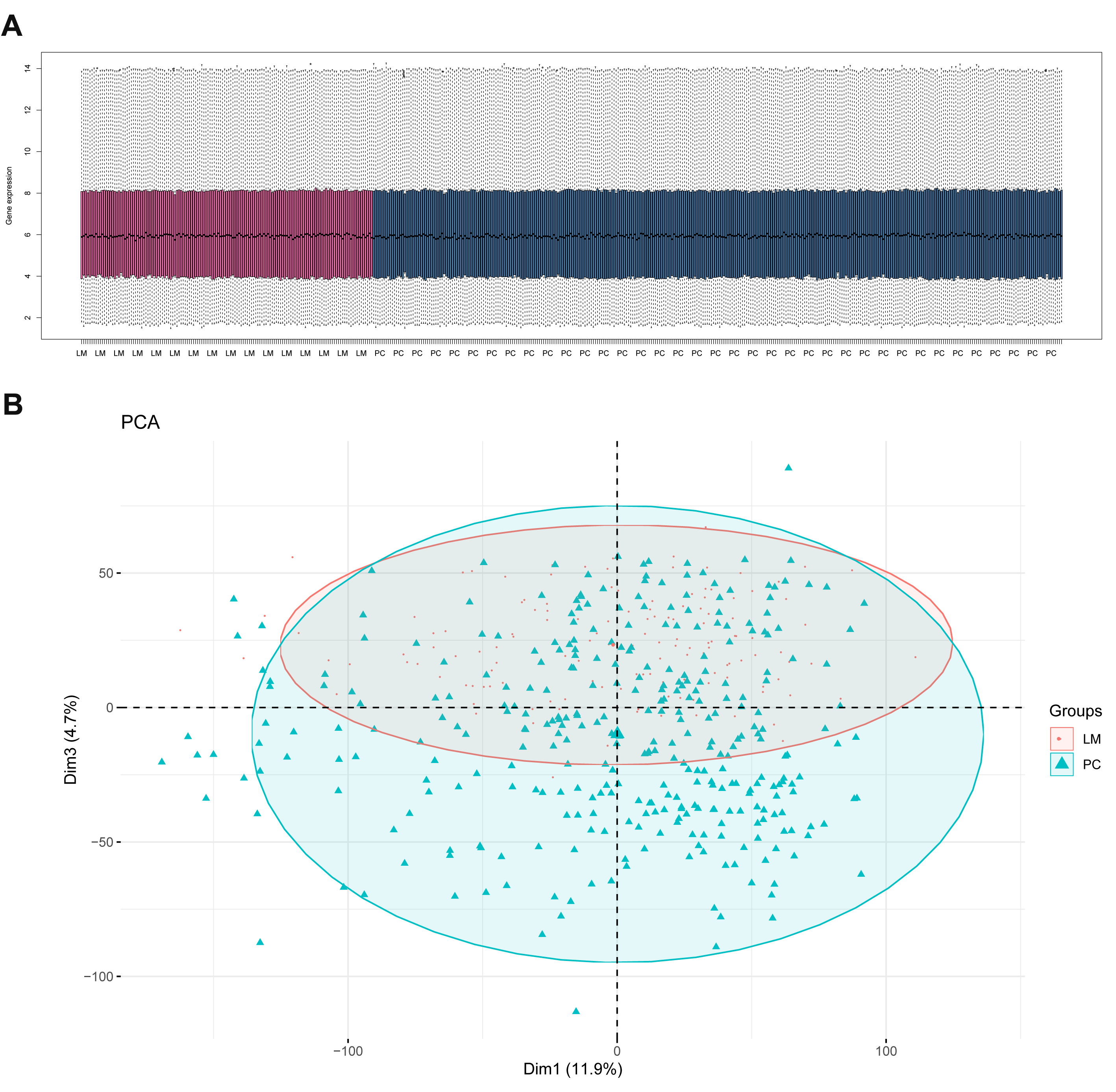

Supplement: Supplementary file 2 — Figure S2. [file CAM4-9-6667-s002.TIF]

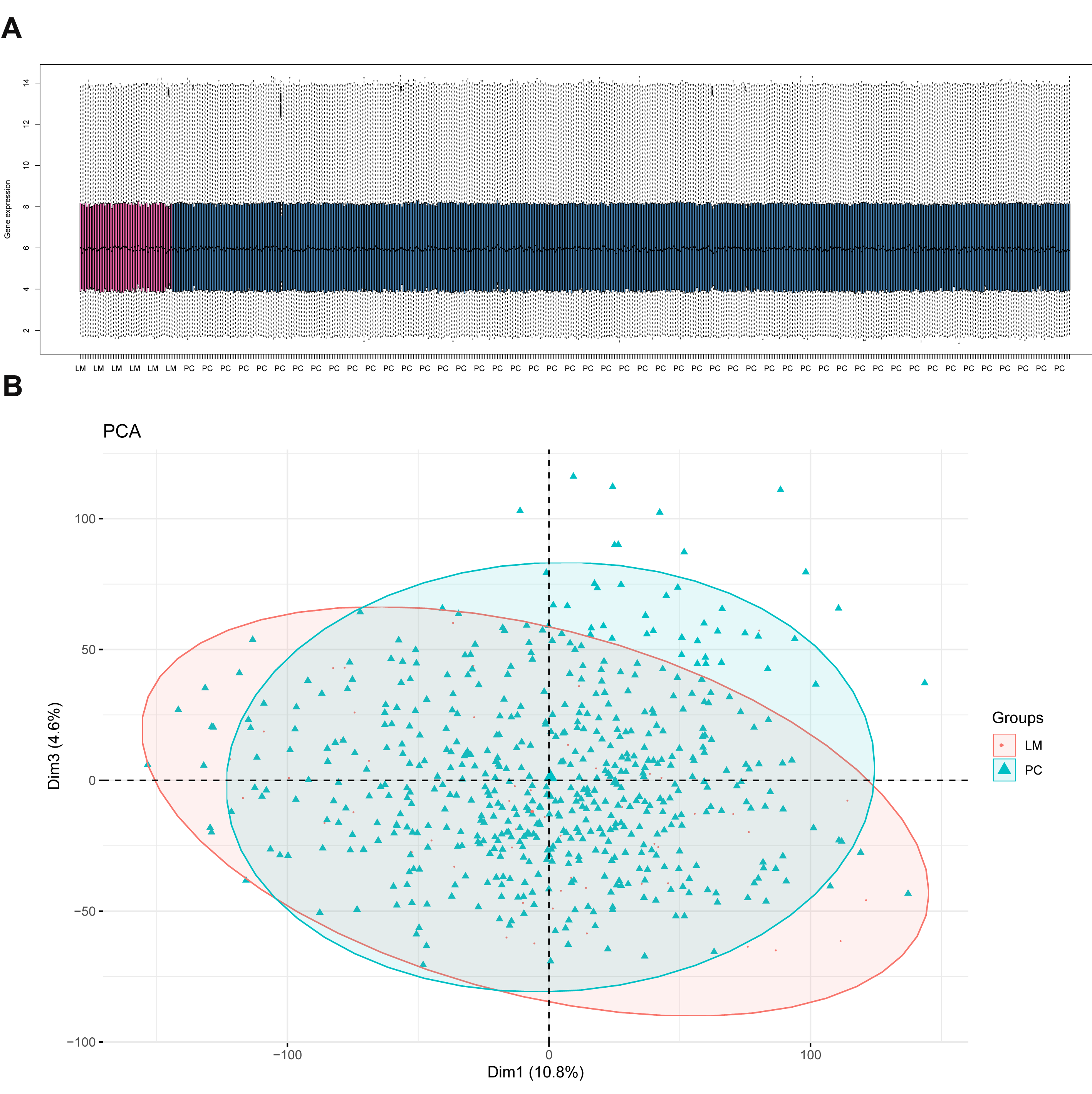

Supplement: Supplementary file 3 — Figure S3. [file CAM4-9-6667-s003.tif]

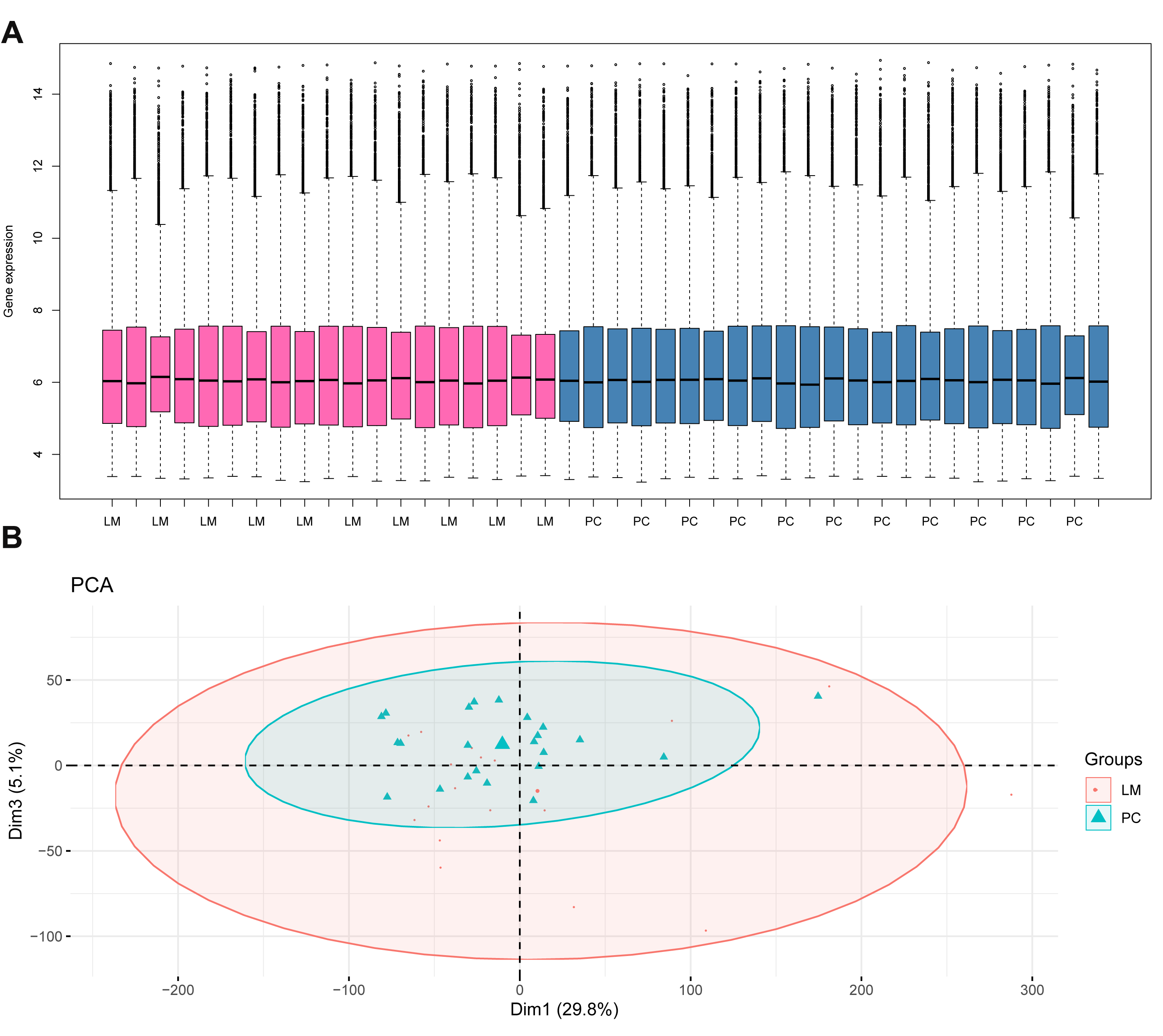

Supplement: Supplementary file 4 — Figure S4. [file CAM4-9-6667-s004.tif]
