# Supplementary material for: Predicting biomarkers from classifier for liver metastasis of colorectal adenocarcinomas using machine learning models
Source: Cancer Med. 2020 Jul 24;9(18):6667–78. doi: 10.1002/cam4.3289 (PMC7520257; doi:10.1002/cam4.3289)
Supplement: Supplementary file 5 — Data S1. [file CAM4-9-6667-s005.docx]

{ "cells": [ { "cell_type": "code", "execution_count": 1, "metadata": {}, "outputs": [ { "data": { "text/plain": [ "'G:\\\\work\\\\1116_19ZWD124F\\\\data\\\\model_L\\\\model'" ] }, "execution_count": 1, "metadata": {}, "output_type": "execute_result" } ], "source": [ "pwd" ] }, { "cell_type": "code", "execution_count": 2, "metadata": {}, "outputs": [], "source": [ "import io\n", "import sys\n", "import time\n", "from warnings import filterwarnings\n", "import matplotlib as mpl\n", "import matplotlib.pyplot as plt\n", "import numpy as np\n", "import pandas as pd\n", "import seaborn as sns\n", "import scipy\n", "import sklearn\n", "#from sklearn.metrics import plot_roc_curve\n", "import sklearn.metrics as mtr\n", "from scipy import stats\n", "from sklearn.discriminant_analysis import LinearDiscriminantAnalysis\n", "from sklearn.ensemble import RandomForestClassifier\n", "from sklearn.ensemble import GradientBoostingClassifier\n", "from sklearn.ensemble import ExtraTreesRegressor\n", "from sklearn.linear_model import LogisticRegression\n", "from sklearn.svm import SVC\n", "from sklearn.neural_network import MLPClassifier\n", "from sklearn.feature_selection import RFE,RFECV\n", "from sklearn.feature_selection import VarianceThreshold\n", "from sklearn.model_selection import StratifiedKFold, train_test_split, KFold\n", "from sklearn.metrics import roc_curve, auc\n", "#from sklearn.pipeline import make_pipeline,Pipeline\n", "from sklearn.preprocessing import StandardScaler\n", "#from matplotlib.backends.backend_pdf import PdfPages\n", "from catboost import CatBoostClassifier,CatBoostRegressor,cv,Pool\n", "#import hyperopt" ] }, { "cell_type": "code", "execution_count": 2, "metadata": {}, "outputs": [], "source": [ "all_time_start = time.time()" ] }, { "cell_type": "code", "execution_count": 3, "metadata": {}, "outputs": [], "source": [ "mcc_data = pd.read_csv ('./liver_mcc_expr.txt',sep='\\t',header=0) " ] }, { "cell_type": "code", "execution_count": 4, "metadata": {}, "outputs": [ { "data": { "text/html": [ "

\n", "
